# Automatic Calibration of the Step Length Model of a Pocket INS by Means of a Foot Inertial Sensor

**DOI:** 10.3390/s20072083

**Published:** 2020-04-07

**Authors:** Dina Bousdar Ahmed, Estefania Munoz Diaz, Juan Jesús García Domínguez

**Affiliations:** 1 German Aerospace Center (DLR), Institute of Communications and Navigation, 82234 Oberpfaffenhofen, Germany; estefania.munoz@dlr.de; 2 Electronics Department of the University of Alcalá, 28805 Alcalá de Henares, Spain; jjesus.garcia@uah.es

**Keywords:** pedestrian localization, wearables, inertial navigation, IMU, step and heading, strapdown, parameter estimation, evaluation, ground truth

## Abstract

All non-foot-mounted inertial localization systems have a common challenge: the need for calibrating the parameters of the step length model. The calibration of the parameters of a step length model is key for an accurate estimation of the pedestrian’s step length, and therefore, for the accuracy of the position estimation. In a previous work, we provided a proof of concept on how to calibrate step length models with a foot inertial navigation system (INS), i.e., an INS based on an inertial measurement unit (IMU) mounted on the upper front part of the foot. The reason is that the foot INS does not require calibration thanks to the implementation of the strapdown algorithm. The goal of this article is to automatically calibrate the parameters of a step length model of the pocket INS by means of the foot INS. The step length model of the pocket INS has two parameters: the slope and offset of a first-order linear regression that relates the amplitude of the thigh pitch with the user’s step length. Firstly, we show that it is necessary to estimate the two parameters of the step length model. Secondly, we propose a method to automatically estimate these parameters by means of a foot INS. Finally, we propose a practical implementation of the proposed method in the pocket INS. We evaluate the pocket INS with the proposed calibration method and we compare the results to the state of the art implementations of the pocket INS. The results show that the proposed automatic calibration method outperforms the previous work, which proves the need for calibrating all the parameters of the step length model of the pocket INS. In this work, we conclude that it is possible to use a foot INS to automatically calibrate all parameters of the step length model of the pocket INS. Since the calibration of the step length model is always needed, our proposed automatic calibration method is a key enabler for using the pocket INS.

## 1. Introduction

Applications based on inertial localization systems have extended from localization in shopping malls or museums [1,2], to safety-critical applications, e.g., tracking a fire fighter’s position [3]. In either case, the trend is to implement the inertial localization system in a smartphone or in a wearable. A wearable is a device with embedded sensors and a processing unit which can be carried out by attaching it to the body, e.g., a smart watch, or by integrating it in the clothes.

In comparison to smartphones, wearables offer an advantage: they can be integrated within the clothes. In fact, the miniaturization of sensors, e.g., inertial sensors, will allow for future smart clothing. The prospect of wearable devices and smart clothing spreading in the market opens up a new alternative for developing inertial localization systems. More specifically, we envision the possibility of developing new inertial localization systems that combine measurements from inertial measurement unit (IMU) placed on different body parts.

The fusion of measurements from IMUs mounted on different body locations may be a solution to the current challenges of inertial localization [4,5]. Some of the challenges are related to the specific body location where the IMU is mounted. For instance, a wrist-mounted inertial localization system needs to cope with the fact that the motion of the arm is detached from the motion of the body. Therefore, it is challenging to track the pedestrian’s position when the hand is not swinging [6,7]. Another challenge of inertial localization systems is related to the integration of the errors in the IMU measurements. For example, the integration of the bias in the turn rate measurements causes the error in the attitude to accumulate over time [8].

There is a specific challenge associated to all non-foot-mounted inertial localization systems. They implement the so-called step&heading algorithm [9]. In this algorithm, the position is estimated in two steps. The first one is the estimation of the pedestrian’s heading. The second one is the estimation of the pedestrian’s step length. There are different alternatives to estimate the step length, for instance:

- by means of a step length model, which is an equation that relates observable parameters, e.g., the acceleration [10], with the step length. The advantage of this alternative is that we can gain an understanding of how the observable parameters relate to the step length model. Moreover, the implementation of step length models has a low computational complexity and therefore can be implemented in wearables or smartphones. The disadvantage of this alternative is that the step length model contains parameters which need to be adapted or tuned to the pedestrian.
- by means of leg kinematics, which is a method that develops a kinematic model of the human leg and uses kinematic equations to iteratively detect steps and estimate the step length between two consecutive steps [11]. The advantage of this alternative is that it is possible to incorporate motion constraints in the kinematic model. The disadvantage is that the motion constraints may lead to unexpected results. In addition, the development of a kinematic model is a complex process.
- by means of machine learning methods, which take observable signals like the acceleration and turn rate and the associated step length and train an algorithm to estimate the step length [12]. The advantage of this alternative is that the machine learning method needs to be trained only once. The disadvantage is that it is necessary to collect a large data set, e.g., hundreds of users, to train the network. Moreover, some machine learning algorithms are computationally expensive and thus cannot be implemented in wearables or smartphones.

In this work, we focus on the first alternative to estimate the step length, i.e., by means of a step length model. As indicated above, step length models have user-specific parameters that need to be tuned to every user. We use the term calibration to refer to this tuning process. What is more, we use the expression *calibration of the step length model* to refer to the estimation of the user-specific constants of the step length model.

The calibration of the step length model is key for an accurate estimation of the pedestrian’s step length. The calibration reduces the error accumulation in the position estimation of non-foot-mounted inertial localization systems. Let us mention here that the error accumulation in the position estimation of the step&heading approach has two sources: the error due to the estimation of the step length and the error due to the heading drift. The latter is the error accumulation in the heading estimation [13].

We distinguish two main approaches in the state of the art to calibrate step length models:

- the use of trained parameters [14], which is the most common calibration approach. These parameters are learned by training the step length model with the data from a set of users. The main challenge of this alternative is that the data set has to be large and varied in order for the parameters to be meaningful. A large data set refers to the number of users, e.g., a data set with data from a few hundred users. To the best of our knowledge, there is no work that recommends a minimum number of volunteers for such a training. A varied data set refers to the fact that the data should be from users of different gender, height, physical constitution, etc. The disadvantage of this calibration method is that it fails to optimally characterize the step length model to a specific user. Thus, the step length model will have a systematic error in its estimation.
- manual calibration [15]. In this method, the user is required to walk a predefined known distance. Then, the user or an external operator estimates an average step length by dividing the predefined distance by the number of steps. Finally, the model parameters are calibrated to estimate the average step length. The shortcoming of this alternative is that it is prone to errors, if the person who carries out the calibration is not familiar with the system. In addition, it is necessary to measure a predefined distance, which may be another source of errors. Manual calibration can be carried out without the need to manually measure distances. To that end, maps can be used to calculate the distance that the user walks [16]. The disadvantage of this alternative is the reliance on the availability of maps, which may not be available or may contain wrong information.

To the best of our knowledge, there are several challenges regarding the calibration of step length models. Three of these challenges are the need for calibrating or re-calibrating when:

- the pedestrian uses the inertial localization system for the first time,
- the IMU is placed on the expected body location, e.g., the upper thigh, but the exact position of the IMU is shifted with respect to the last time the calibration was carried out or
- the IMU slips during the walk.

All these situations make the calibration of step length models a tedious process. We believe there is an opportunity of tackling the challenge of automatically calibrating step length models thanks to the increasing popularity of wearables devices, especially smart clothing. We think it is of interest to assess the potential of using a foot IMU, i.e., an IMU mounted on foot or integrated in the shoes, to calibrate the step length models of non-foot-mounted inertial localization systems. The reason is that, through a foot IMU, it is possible to estimate increments in distance without the need of a step length model. To that end, it is only necessary to implement the strapdown algorithm with the zero-velocity update (ZUPT) [8]. In fact, since the ZUPT can also be applied with an ankle-mounted IMU, such body location could also be used to estimate increments in distance.

In [17], we provided a proof of concept on how to automatically calibrate step length models with a foot inertial navigation system (INS). We considered the step length model of a pocket INS, which is an inertial localization system based on an IMU mounted on the upper thigh. The disadvantage of the proposed method is that it is not valid for certain users, which leads to an increased error in the position accuracy.

The goal of this article is to extend the work in [17] to develop a method to automatically calibrate the parameters of the step length model. In particular, we have the following objectives:

- identify if it is necessary to calibrate all the parameters of the step length model of an IMU mounted on the upper thigh,
- develop an automatic calibration method based on a foot IMU,
- quantitatively evaluate the performance of the pocket INS with the proposed calibration method and compare it to the performance of the pocket INS without automatic calibration.

## 2. Materials and Methods

In this section, we describe the method to automatically calibrate the step length model of a pocket INS with a foot IMU. First, we describe two fundamental concepts on which the automatic calibration is based. Then, we show that it is necessary to calibrate all the parameters of the step length model of the pocket INS for certain users. Finally, we present the method to automatically calibrate the step length model of the pocket INS by means of the foot INS.

**Figure 1:**
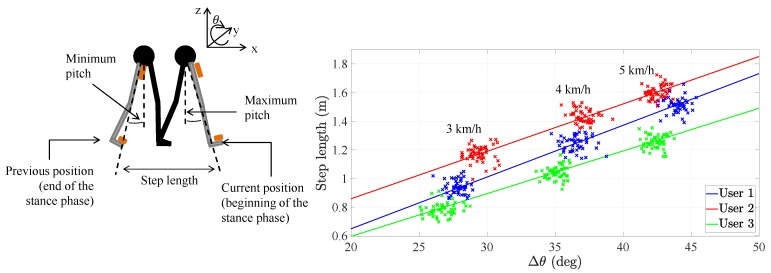
(**Left**) Visualization of the maximum and minimum of the pitch *θ* of the pocket inertial navigation system (INS) while walking as well as the user’s step length. The stance phase is the gait phase during which the foot is in contact with the ground. The orange boxes are the inertial measurement units (IMUs). (**Right**) Relationship between the pitch amplitude of the pocket INS and the step length for three different users. The point clouds depend on the walking speed of the user.

For the remainder of this paper, we will consider that:

- two IMUs are fixed on the same leg of the user and they do not move during the walk, see left picture in Figure 1,
- one IMU is mounted on the upper-thigh, and
- one IMU is mounted on the front part of the foot.

### 2.1. Fundamental Concepts

There are two fundamental concepts to take into account prior to describing the method to automatically calibrate step length models. The first one is that the step length model depends on the body location of the IMU. Since we are using a pocket IMU, we will describe the associated step length model in order to understand what the model represents as well as its parameters. The second one is the proof of concept presented in [17] by which we show that a foot INS can be used to automatically calibrate step length models.

#### 2.1.1. Step Length Model of a Pocket INS

In pedestrian dead reckoning, there are different step length models that relate an observable signal, e.g., the norm of the acceleration, with the step length [10]. Although we focus on the step length model of the pocket INS, the automatic calibration method based on a foot INS is extensible to the step length model of other inertial localization systems.

The derivation of the step length model of the pocket INS is detailed in [18] and references therein. For completeness, we present here a summary of the step length model. Munoz et al. observed that the relationship between the user’s step length and amplitude of the thigh pitch is approximately linear for different walking speeds [18], see Figure 1. Therefore, the step length model of the pocket INS relates the step length $s^{k}$ with the amplitude of the thigh pitch $\Delta \theta^{k}$ through a first-order linear regression [18], such that:

(1) $$s^{k}=a\cdot \Delta \theta^{k}+b+e^{k},$$

where the slope *a* and the offset *b* are user-specific parameters that have to be estimated during a calibration phase. More specifically, the slope *a* depends on the user’s physiology and its value is constant over time. The offset *b* depends on the walking style of the person and, therefore, its value may change over time. $e^{k}$ is an unobservable random variable that represents the error in the first-order linear regression model [19]. The super-index *k* refers to the *k*-th step and the pitch angle *θ* is the rotation around the y-axis and therefore represents the opening angle of the leg, see Figure 1.

Figure 1 exemplifies the relationship of Equation (1) for three different users during walking. The walking speed conditions the step length $s^{k}$ and the pitch amplitude $\Delta \theta^{k}$ of the pocket IMU. In fact, the higher the walking speed, the higher these values are. From Figure 1, it can be seen that different users have approximately the same slope *a*. In contrast, the offset *b* differs significantly among users. The study in [18] states that the value of the offset *b* depends on the walking style of the person.

The previous results need to be taken into account because they lead to two possible alternatives of calibrating the step length model in Equation (1):

- The first alternative is the offset calibration, which implements the universal slope *a* and calibrates the offset *b* to every user. The universal slope is the value obtained after training the parameters on a set of users, namely 0.05 m/° [15]. This calibration method assumes that the user’s slope equals the universal slope, which may not be a valid assumption for certain users.
- The second alternative is the full calibration, which estimates the user’s optimal slope and optimal offset by following the methodology described in [18]. This calibration method is costly in time and resources. Firstly, it is necessary to request the user to actively change the walking speed in order to obtain values along the line in Figure 1. Secondly, it is necessary to estimate the parameters of the step length model in a post-processing stage. Finally, it may still be necessary to adjust the offset *b* if the inertial sensor is not placed on the same position along the leg [17].

Therefore, for practical reasons, Munoz proposes to follow the offset calibration of the step length model [15]. Following such an alternative, next section summarizes our proposal on how to implement the offset calibration automatically. The full calibration is the main contribution of this article and will be presented later on.

#### 2.1.2. Automatic Offset Calibration

The proof of concept to automatically calibrate one parameter of the step length model of the pocket INS is detailed in [17]. For completeness, we present a summary, which estimates the offset *b* of the step length model of the pocket INS.

The automatic offset calibration estimates the offset *b* of Equation (1) when the slope *a* is set to the universal value 0.05 m/°. The calibration method uses a foot INS [9] which is mounted on the same leg as the pocket INS. The reason for using the foot INS is that it implements the strapdown algorithm together with the ZUPT update [8]. Therefore, the foot INS provides a measurement of the user’s step length without the need of a model. Moreover, the accuracy of the user’s step length estimated with the foot INS is at least 20 cm per step [5], which is smaller than the size of an adult’s foot. Thus, we consider the foot INS presented in [5] to have an accuracy good enough for our goal.

Let $s_{f}^{k}$ be the user’s step length estimated by the foot INS and $\Delta \theta^{k}$ be the amplitude of the pitch estimated by the pocket INS. It can be proven that the offset $b^{k}$ that optimizes the model of Equation (1) is iteratively estimated as:

(2) $$b^{k}=b^{k-1}\cdot \frac{k-1}{k}+\frac{s_{f}^{k}-a\cdot \Delta \theta^{k}}{k},$$

where *k* refers to the *k*-th step.

**Figure 2:**
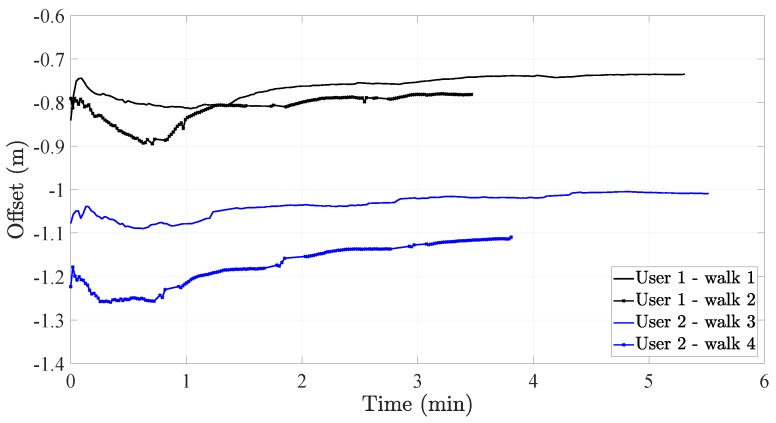
Recursive estimation of the offset of the step length model according to the automatic offset calibration method. The example is given for two users and two tests for each user.

When the slope *a* is adapted to the user, then the offset estimated by Equation (2) converges after approximately 2 min. An example is given in Figure 2 which presents the offset estimation during four different walks for two different users. As expected, different users have different offset estimations. In the figure, an interesting phenomenon can be observed. The offset estimations of User 2 take two different values during each walk. The reason may be that the pocket IMU was not placed at the exact same location on each day. For User 1, the two offset estimations differ less than for User 2, which is why we consider them to be approximately the same.

The advantage of the calibration through Equation (2) is its low computational complexity. Moreover, the automatization of the calibration method simplifies the use of the pocket INS. In contrast to the state of the art methods, the user is not aware of the need for calibration with our proposed method nor the fact that the step length model of the pocket INS is being calibrated.

### 2.2. Analysis of the Need for Full Calibration of the Step Length Model

When the step length model of Equation (1) was first proposed [18], Munoz et al. pointed out that the slope of the model remained approximately constant among users. This constant value is the universal value mentioned above, i.e., 0.05 m/°. The followup work in [15] shows that some users might not have a slope similar to the universal one. This fact may be caused by the user’s height, the walking style or any other aspect that the author could not specify. Independently of the cause, the highlight is that the slope *a* of the step length model is not optimal to certain users and thus, the slope *a* needs to be calibrated.

**Figure 3:**
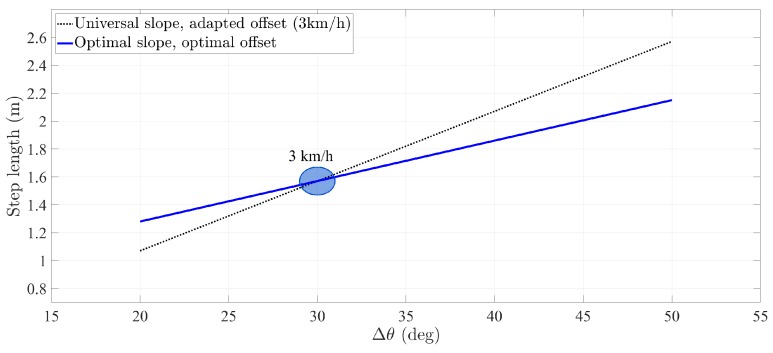
Comparison of the step length model adapted to the same user with two different set of parameters. The first set consists of the universal slope and the adapted offset when the user walks at 3 km/h. The second set is the optimal slope and the optimal offset that characterize the user.

The fact that a user’s optimal slope differs from the universal slope is, in principle, not noticeable with the calibration method of Section 2.1.2. This statement holds as long as the user walks at a constant speed. Let us analyze the example in Figure 3. While walking at a certain speed, e.g., 3 km/h, a user has a specific amplitude of the thigh pitch $\Delta \theta^{k}$ and associated step length $s^{k}$. These values are distributed along the circle depicted in Figure 3. For these given pairs of step length–pitch amplitude, the step length model of Equation (1) can be adapted with different sets of parameters. For instance, the universal slope and adapted offset, black line, or the optimal slope and optimal offset, blue line.

**Figure 4:**
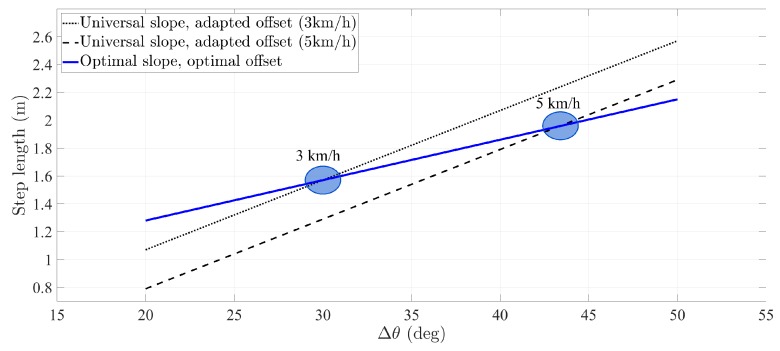
Effect of calibrating only the offset when the universal slope differs from the user’s optimal slope. When the user walks at different speeds, the calibrated offset changes, e.g., the offset is 0.07 m at 3 km/h, whereas the offset equals −0.21 m at 5 km/h.

In contrast, if the user walks faster or slower, the change in speed will be reflected in a change of the offset *b* estimated according to Equation (2). Figure 4 is an example of the different offset estimations at different walking speeds. These different estimations are an indication that the universal slope is not adapted to the user. In such a case, a continuous offset calibration with Equation (2) would suffice to adapt the step length model to the user continuously over time. Nevertheless, a continuous calibration would result in an inefficient procedure.

The users who belong to the case described by Figure 3 and Figure 4 require a full calibration of the step length model, i.e., the estimation of both the optimal slope and optimal offset. The automatic calibration method for such cases is described in detail in the following section.

### 2.3. Automatic Full Calibration of the Step Length Model

In order to estimate the optimal slope and optimal offset of the user, let us consider again the step length model of Equation (1):

(3) $$s_{f}^{k}=a\cdot \Delta \theta^{k}+b+e^{k}.$$

The automatic calibration method aims at estimating the predefined constants *a* and *b* that minimize the error $e^{k}$ of the step length model. Let us denote by *a_o_* and *b_o_* the optimal slope and optimal offset, respectively, that minimize the error in Equation (3). By means of the least squares method [20], the optimal offset and optimal slope are estimated as follows:

(4) $$a_{o}=\frac{n\cdot \sum_{k=1}^{n}\Delta \theta^{k}\cdot s_{f}^{k}-\sum_{k=1}^{n}\Delta \theta^{k}\cdot \sum_{k=1}^{n}s_{f}^{k}}{n\cdot \sum_{k=1}^{n}{\left(\Delta \theta^{k}\right)}^{2}-{\left(\sum_{k=1}^{n}\Delta \theta^{k}\right)}^{2}},$$

(5) $$b_{o}=\frac{\sum_{k=1}^{n}{\left(\Delta \theta^{k}\right)}^{2}\cdot \sum_{k=1}^{n}s_{f}^{k}-\sum_{k=1}^{n}\Delta \theta^{k}\cdot s_{f}^{k}\cdot \sum_{k=1}^{n}\Delta \theta^{k}}{n\cdot \sum_{k=1}^{n}{\left(\Delta \theta^{k}\right)}^{2}-{\left(\sum_{k=1}^{n}\Delta \theta^{k}\right)}^{2}},$$

where *n* is the total number of steps since the beginning of the walk.

These two equations have been used in an example to estimate the optimal parameters of a user. The example compares the regression line with two sets of parameters; on the one hand, the optimal slope and optimal offset, on the other hand, the universal slope and the adapted offset. Moreover, the example considers three walking speeds: low speed, medium speed and high speed. Taking the experiment of Munoz as a reference [15], we consider a slow walking speed to be 5 km/h, a medium walking speed to be 6 km/h and a fast walking speed to be 7 km/h.

**Figure 5:**
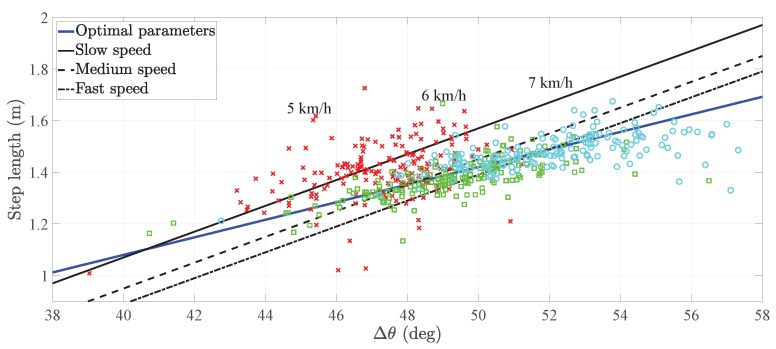
Comparison of the different step length models of a user whose optimal slope differs from the universal one. The clouds indicate the pairs of step length–pitch amplitude used to train the model parameters. The cross marks, square marks and circle marks are associated to 5 km/h, 6 km/h and 7 km/h, respectively.

Figure 5 shows that, at different walking speeds, the offset calibration estimates different values. More specifically, the offset difference between the lowest speed and highest speed is 18 cm. In this example in particular, the user’s optimal slope is $a_{o}=0.022\;m/{}^{\circ}$, whereas the universal slope is 0.05 m/°.

**Figure 6:**
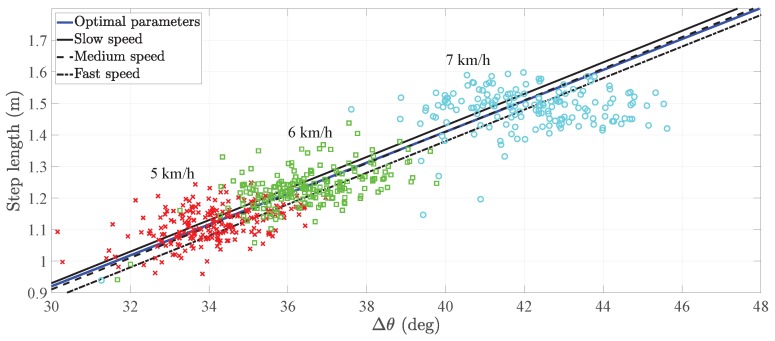
Comparison of the different step length models of a user whose optimal slope is approximately the same as the universal one. The clouds indicate the pairs of step length–pitch amplitude used to train the model parameters. The cross marks, square marks and circle marks are associated to 5 km/h, 6 km/h and 7 km/h, respectively.

Figure 6 is an example of the full calibration where the user’s optimal slope is approximately equal to the universal slope. In particular, the difference in the offset between the lowest speed and highest speed is 5 cm which is approximately 72% smaller than the difference in Figure 5. Therefore, we can assume that the slope is adapted to the user.

An interesting fact to observe in Figure 5 and Figure 6 is that the point clouds are not equally distributed. The distribution is characteristic of each user and responds to the user’s physiology and walking style.

### 2.4. Practical Implementation

**Figure 7:**
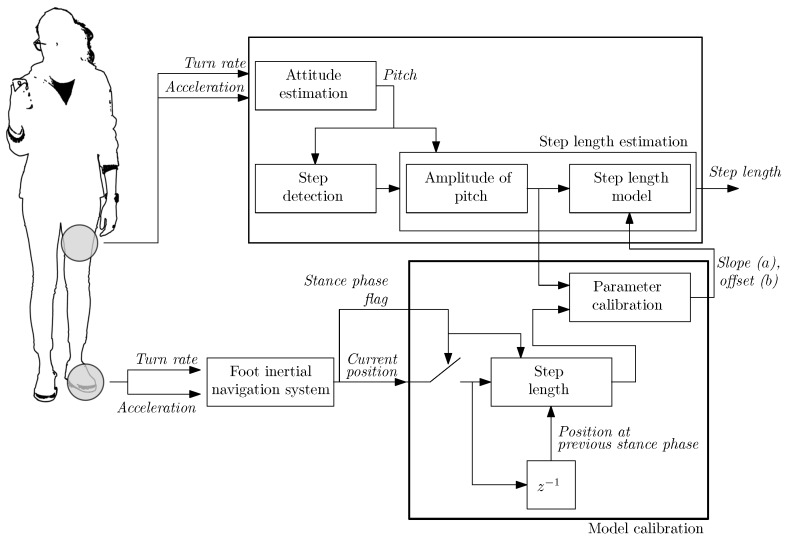
The block diagram of the calibration method is indicated by the thick solid line. The blocks of the pocket INS that are relevant for the calibration method are shown.

Figure 7 presents the block diagram of the calibration method of step length models. In the diagram, only the blocks of the pocket INS that are relevant for the calibration method are shown. In a practical case, the pocket INS follows the implementation described in [15].

In order to carry out the full calibration, the position estimated by the foot INS is sampled upon the detection of the stance phase to estimate the step length, see Figure 1. Then, the estimated step length is input to the *Parameter calibration* block which implements Equations (4) and (5). Finally, the estimated slope and offset are input to the step length model in order to estimate the step length.

## 3. Results

The last goal of this work is to evaluate the proposed method to automatically calibrate the step length model of the pocket INS. To that end, this section describes the evaluation methodology to assess the performance of the pocket INS. Then, the results of the evaluation are presented for the pocket INS with four different calibrations of the step length model.

### 3.1. Evaluation Methodology

Our ground truth system, similar to indoor localization competitions [21,22], is based on ground truth points [4,5]. The location of these points is measured in advance. Then, the user visits these points in a predefined sequence. The inertial localization system is evaluated by comparing its estimated position of a ground truth point to the true position of the ground truth point.

The location of the ground truth points is measured with a laser distance measurer. It has, approximately, centimeter accuracy which is at least one order of magnitude smaller than the expected accuracy of the inertial localization systems under evaluation. This criterion is recommended by the ISO standard [23].

In this work, the inertial localization systems are evaluated with three metrics. The first two are the distance error *e*_d_ and the heading error *e_ψ_*, which are defined as follows:

(6) $$e_{d}=|d_{ij}^{r}-d_{ij}^{w}|,$$

(7) $$e_{\psi }=|\psi_{ij}^{r}-\psi_{ij}^{w}|,$$

where $d_{ij}^{r}$ and $d_{ij}^{w}$ are the true horizontal distance and the estimated horizontal distance between the *i*-th and the *j*-th ground truth points, respectively. Similarly, $\psi_{ij}^{r}$ and $\psi_{ij}^{w}$ are the true angle and estimated angle between the *i*-th and the *j*-th ground truth points. |·| denotes the absolute value of the argument. *e*_d_ and *e_ψ_* are representative of the distance and heading error only if the trajectory between the *i*-th the *j*-th point is straight. This consideration is taken into account in the design of the experiments.

The third metric is the height error *e*_h_, which is defined as follows:

(8) $$e_{h}=\frac{\left|h_{i}^{r}-h_{i}^{w}\right|}{\Delta h_{i}^{r}},$$

where $h_{i}^{r}$ and $h_{i}^{w}$ are the true height and the estimated height of the *i*-th ground truth point. |·| denotes the absolute value of the argument. $\Delta h_{i}^{r}$ is the total height change at the *i*-th ground truth point, that is:

(9) $$\Delta h_{i}^{r}=\sum_{j=0}^{i}\left|h_{j}-h_{j-1}\right|,$$

where $h_{j}$ is the height of the *j*-th ground truth point. Equation (8) represents a height error normalized to the total change in height. For instance, a height error *e*_h_ equal to 0.1 m/m tells us that the inertial localization system makes an error of 10 cm in a height change of 1 m.

**Figure 8:**
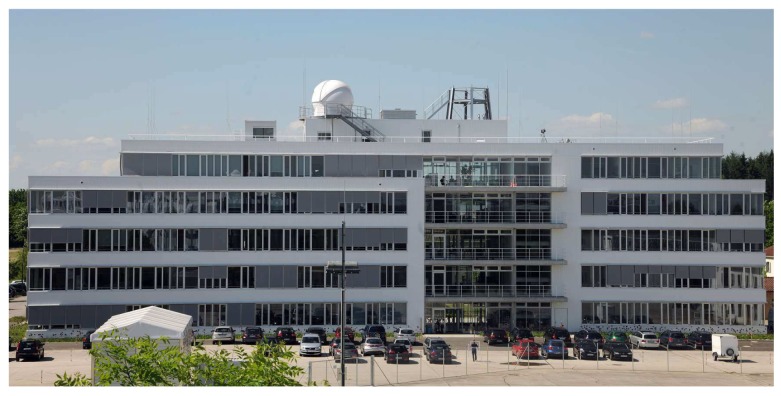
Five-storey building where the experiments took place.

**Figure 9:**
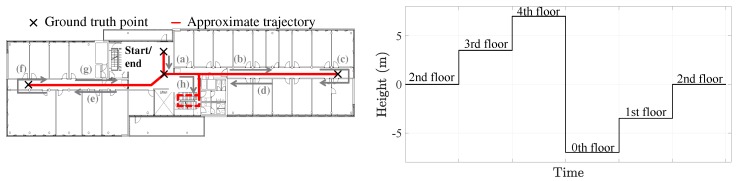
(**Left**) Approximate 2D trajectory, the users started at the indicated point and walked in the order indicated by the letters (a)–(h). The dashed lines on the floor plan indicate the stairs. (**Right**) Height profile followed by the users. The height difference between two consecutive floors is 3.5 m.

The data set was collected during a set of experiments which took place in a five-storey building, see Figure 8. During the experiments:

- The users visited each of the five floors in the sequence indicated by the height profile in Figure 9, i.e., they started in the second floor, then proceeded to the third floor by taking the stairs, etc.
- The users always took the stairs during the experiments.
- In each floor, the users walked the trajectory indicated on the left picture of Figure 9.

Each user carried out each walk in the following phases:

1. the user starts on the second floor, at the point indicated on the right picture of Figure 9,
2. the user follows the sequence indicated by the letters (a)–(h) on the left picture of Figure 9,
3. the user takes the stairs to the next floor. The stairs are indicated by the dashed lines on the left picture of Figure 9. The floor sequence is indicated by the height profile on the right picture of Figure 9,
4. on the new floor, the users follow the trajectory (b)–(h). For each floor, the user repeats phases 3 and 4 until the user is back to the end point on the second floor.

Regarding the ground truth points, the users visited three points on each floor, see Figure 9. The users were instructed to stop 2 s to 3 s at each ground truth point to signal that they reached one. This approach is also followed in indoor localization competitions [24]. Finally, users of different heights and different ages participated in the experiment. Each user repeated the aforementioned trajectory twice, and each trajectory lasted approximately 15 min to 20 min. The users were equipped with two IMUs mounted on the upper thigh and the front part of the foot, respectively. The IMUs were fixed to the associated positions with straps that assured that the devices would stay fixed during the experiments. The IMUs are measurement units from Xsens [25] whose noise characteristic are given in [4].

**Figure 10:**
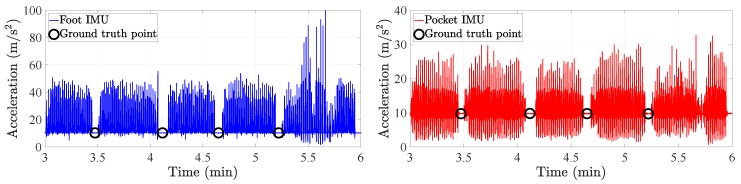
Identification of the stop at the ground truth points. The norm of the acceleration vector is presented for the foot IMU (**left**) and the pocket IMU (**right**).

The summary of the experiments is given in Table 1. The outcome of the experiments is the acceleration vector and turn rate vector from both the pocket IMU and the foot IMU of each user. In addition, the ground truth points are identified by detecting when the user stopped at each ground truth point. This detection is done by analyzing the norm of the acceleration vector of either IMU. An example is given in Figure 10, where the acceleration norm of both the foot IMU and the pocket IMU is represented. We can see how the stop at the ground truth points are clearly observed by the periods of constant acceleration.

### 3.2. Results and Discussion

The evaluation presented in this section has two goals. Firstly, we aim at evaluating the pocket INS with the full calibration described in this article. Secondly, we aim at comparing the performance of the full calibration with the state of the art calibration methods of the step length model. We consider three state of the art calibration methods of the step length model:

- in the first one, the step length model implements the universal parameters [15]. In the following, we refer to this implementation of the pocket INS as *universal parameters*.
- in the second one, the step length model is manually calibrated to each user [15]. In the following, we refer to this implementation of the pocket INS as *manual calibration*.
- in the third one, only the offset of the step length model is automatically calibrated while the slope is set to the universal value, namely 0.05 m/°, see Section 2.1.2. In the following, we refer to this implementation of the pocket INS as *offset calibration*.

In order to evaluate the pocket INS with the full calibration we have followed two steps. Firstly, we have estimated the optimal parameters of the users during a dedicated walk where the users were instructed to change their walking speed as depicted in Figure 5 and Figure 6. In order to estimate a user’s optimal parameters, we have followed the implementation proposed in this article, see Figure 7. The optimal parameters estimated for four different users are given in Table 2. Secondly, we have evaluated the pocket INS with two sets of parameters for the step length model:

- the first set of parameters comprises the ones indicated in Table 2. In the following, we refer to this implementation of the pocket INS as *full calibration*.
- the second set of parameters comprises the user’s optimal slope, estimated with the proposed method, whereas the offset is calibrated with Equation (2). For the implementation of Equation (2), we have set the slope *a* to the user’s optimal value, see Table 2. In the following, we refer to this implementation of the pocket INS as *hybrid calibration*.

The results of the evaluation are given in Table 3, which shows that all the implementations of the pocket INS have the same heading error. This result is expected since the calibration method effects only the step length estimation of the step&heading algorithm. The same applies to the height error, where the vertical displacement is not influenced by the step length model [15]. That is, the effect of the proposed calibration methods on the heading error and height error are consistent with the expectations.

The key metric in this evaluation is the distance error. Table 3 shows not only the mean and standard deviation of the distance error *e*_d_ but also its third quartile. Such metric is used commonly in indoor localization competitions to compare different systems [24]. The calibration with universal parameters has the highest distance error because it is the one that worst models the physiology of an user.

**Figure 11:**
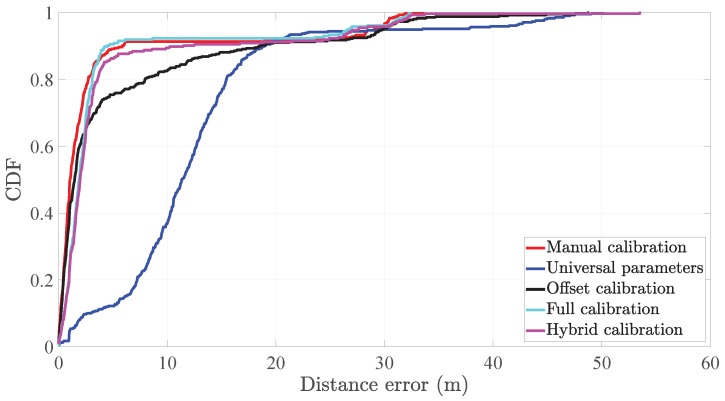
Cumulative distribution function (CDF) of the distance error *e*_d_ of the pocket INS with different configurations.

Table 3 indicates that the full calibration outperforms both the offset calibration and the hybrid calibration. This result shows the benefits, in distance accuracy, of calibrating both parameters of the step length model. The cumulative distribution function (CDF) of Figure 11 supports this result.

The mean distance error of the hybrid calibration outperforms the mean distance error of the offset calibration by 15%. Yet, the third quartile of the distance error of the hybrid calibration outperforms the same metric of the offset calibration in 34%. In fact, the CDFs of the distance error in Figure 11 depict a clear advantage of the hybrid calibration over the offset calibration.

By comparing the full calibration and the hybrid calibration, we observe that the full calibration outperforms the hybrid calibration by a small margin, i.e., 15% regarding the mean distance error *e*_d_ and 10% regarding the third quartile of the distance error *e*_d_. Moreover, the CDFs of the distance error *e*_d_ of both systems are approximately similar.

The main difference between the full calibration and the hybrid calibration is in their practical implementation. The full calibration requires the user to actively participate in the calibration process by changing the walking speed. Such step is necessary to estimate the parameters of the step length model that represent the user’s physiology. If the IMU moves during the walk, a new full calibration is necessary. Although such process leads to the same optimal slope, the new estimated offset is different from the one estimated initially.

In contrast, the hybrid calibration assumes that once the user’s optimal slope is estimated, only a calibration of the offset is necessary. As shown in previous works [15], the user’s optimal slope remains constant while the offset may vary depending on the walking style of the person. Therefore, the hybrid calibration combines the benefits of both the full calibration and the offset calibration:

- with respect to the full calibration, the hybrid calibration benefits from using the user’s optimal slope which clearly outperforms the offset calibration, see Table 3.
- with respect to the offset calibration, the hybrid calibration benefits from the fact that it is only necessary at the beginning of the walk and it is a process transparent to the user [17].

As expected, the benefits of the hybrid calibration come at a cost of a higher distance error *e*_d_ than the full calibration.

Interestingly, none of the implementations of the pocket INS with the automatic calibration methods outperforms the manual calibration. The reason is that we calibrate the step length model with the foot INS, which has errors as well. Thus, there is a lower bound to the accuracy of the step length model, i.e., the estimation of the step length model cannot outperform the distance estimation of the foot INS. In addition, the step length model has errors due to the model itself. The combination of the latter two error sources explains why the distance error *e*_d_ of the proposed pocket INSs with automatic calibration do not outperform the pocket INS with manual calibration in Table 3.

**Figure 12:**
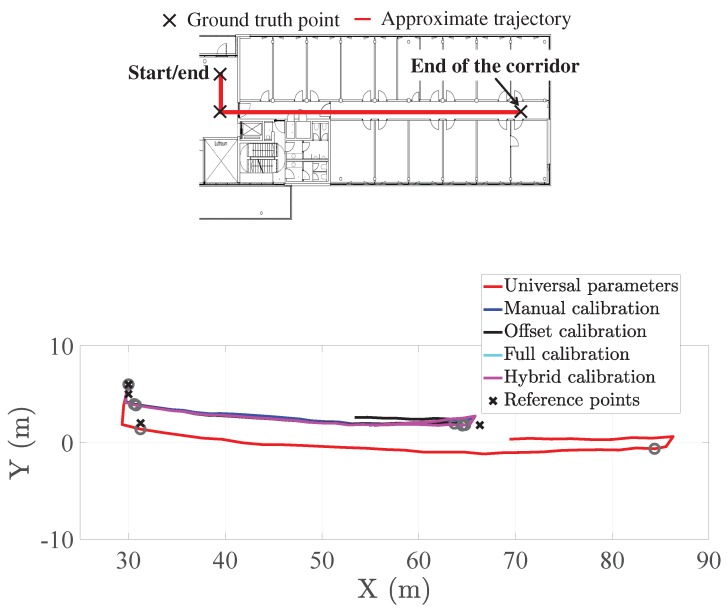
(**Top**) Approximate true trajectory. (**Bottom**) Example odometry of the pocket INS with different calibration methods. The circle marks are the estimated positions of the ground truth points.

We finalize this section with an example of the odometry to show the effect of the calibration method. Figure 12 shows one stretch of a user’s trajectory: from the start to the end of the corridor. The duration of this stretch is approximately 60 s. The odometry estimated by the pocket INS with universal parameters clearly estimates longer distances than any other version of the pocket INS. We appreciate this fact by comparing the true ground truth point at the end of the corridor with the estimated one. The distance overestimation is due to the use of the universal parameters in the step length model. In this example, the parameters do not model the physiology of the user, thus leading to the overestimation of the step length, which leads to an overestimation of the distance.

The remaining versions of the pocket INS have approximately the same accuracy. In fact, we observe that the estimated odometry reaches, approximately, the end of the corridor. The error at the end of the corridor is 1.8 m over a total distance of 35 m, which is the length of the corridor. That is, the error at the end of the corridor is 5% of the length of the corridor.

With the proposed full calibration method, we can estimate the two parameters of the step length model of the pocket INS provided that the user changes the walking speed. The resulting slope is the optimal slope of the user and it only needs to be estimated once. If the IMU moves, the full calibration is necessary again, which means that the user would have to actively participate in the calibration process again by changing the walking speed. In order to ease the calibration process for the user, we propose an alternative hybrid calibration. According to it, the step length model of the pocket INS can be used by setting the slope to the user’s optimal value. Such a value is estimated with the approach proposed in this article. Then, the offset can be calibrated in each walk with Equation (2).

## 4. Conclusions

In this article, we have presented a method to automatically calibrate the step length model of a pocket INS. The step length model has two parameters: the slope and offset of a first-order linear regression which relates the leg aperture with the step length. The proposed method uses a foot INS to estimate the parameters of the step length model that are specific to a user. Moreover, we have evaluated the performance of the proposed method and compared it to the state of the art calibration methods.

There are three main conclusions from the work we have presented. The first one is that there are users whose optimal slope differs from the universal slope, which is the one used by default. For such users, the offset of the step length model changes with the walking speed, hence making the calibration of the offset necessary every time the walking speed changes. The second one is that we can automatically estimate the two parameters of the step length model of the pocket INS by using the output of the foot INS. With our proposed method, we find the user’s optimal parameters for the step length model. The third conclusion is that the full calibration needs to be done only once to estimate the optimal slope of the user. The resulting optimal slope can then be used in the pocket INS as the characteristic value of the user’s and the offset can be calibrated for each walk using the method we have proposed in a previous work.

## 5. Patents

The work presented in this article is related to a patent which is being under consideration at the German Patent Office. The file number is 10 2017 103 607.5.

## Figures and Tables

**Table 1:** Summary of the experiments.

No. of Users	Total Time	Total No. of Ground Truth Points
4	3 h 20 min	295

**Table 2:** Optimal parameters of the step length model estimated with the proposed full calibration method.

User	Slope *a* [m/°]	Offset *b* [m]
User 1	0.0288	0.1
User 2	0.0523	−0.7
User 3	0.0450	−0.5
User 4	0.0437	−0.6

**Table 3:** Evaluation of the pocket INS with different calibration configurations. The performance figures are given as mean ± standard deviation of the error metrics. Q_3_(*e*_d_) is the third quartile of the distance error.

System Description	*e*_d_ [m]	Q_3_(*e*_d_) [m]	*e_ψ_* [°]	*e*_h_ [m/m]
Universal parameters	12.6±8.3	14.9	57.5±49.6	0.6±1.4
Manual calibration	3.4 ± 7.7	2.3	61.1 ± 48.6	0.6 ± 1.4
Offset calibration	5.0±8.4	4.7	57.0±49.4	0.6±1.4
Full calibration	4.0±7.3	2.8	57.7±50.7	0.6±1.4
Hybrid calibration	4.7±8.2	3.1	57.7±50.7	0.6±1.4

## Abbreviations

The following abbreviations are used in this manuscript:

IMU	Inertial Measurement Unit
INS	Inertial Navigation System
ZUPT	Zero-velocity UPdaTe
CDF	Cumulative Distribution Function
